# Hysteretic Behavior of Prestressed Concrete Bridge Pier with Fiber Model

**DOI:** 10.1155/2014/467350

**Published:** 2014-01-22

**Authors:** Wang Hui-li, Feng Guang-qi, Qin Si-feng

**Affiliations:** ^1^State Key Laboratory of Structural Analysis for Industrial Equipment, Dalian University of Technology, Bridge Engineering Research Institute, Dalian University of Technology, Dalian 116023, China; ^2^Bridge Engineering Research Institute, Dalian University of Technology, Dalian 116085, China; ^3^Research Center for Numerical Tests on Material Failure, Dalian University, Dalian 116622, China

## Abstract

The hysteretic behavior and seismic characteristics of the prestressed concrete bridge pier were researched. The effects of the prestressed tendon ratio, the longitudinal reinforcement ratio, and the stirrup reinforcement ratio on the hysteretic behavior and seismic characteristics of the prestressed concrete bridge pier have been obtained with the fiber model analysis method. The analysis show some results about the prestressed concrete bridge pier. Firstly, greater prestressed tendon ratio and more longitudinal reinforcement can lead to more obvious pier's hysteresis loop “pinching effect,” smaller residual displacement, and lower energy dissipation capacity. Secondly, the greater the stirrup reinforcement ratio is, the greater the hysteresis loop area is. That also means that bridge piers will have better ductility and stronger shear capacity. The results of the research will provide a theoretical basis for the hysteretic behavior analysis of the prestressed concrete pier.

## 1. Introduction

In the Kobe earthquake in 1995, about one-fifth of common reinforced concrete piers did not need reconstruction because of excessive residual plastic deformation. Therefore, international scholars made a mass of tests and theoretical researches on high ductility and low-residual displacement pier [1]. Supported by the Japanese Prestressed Concrete Engineering Association, according to the disadvantages of low energy dissipation capacity and poor ductility performance of pure prestressed concrete (PRC) pier and large residual plastic deformation of pure common reinforced concrete pier, De Felice [2] put forward an assumption of the mixed use of common reinforcement and prestressed tendon in a pier. The vertical prestressed tendon can decrease residual plastic deformation of pier. In addition, the common reinforcement can increase ductility capacity and energy dissipation capacity and control the crack of pier body.

Prestressed concrete bridge piers were adopted in many famous bridges [3, 4], such as the Pontchartrain Bridge in USA, Angle Island Bridge in Japan, Montsoreau Bridge in France, and Donghai Bridge and Hangzhou Bridge in China. Research [5–11] showed that adopting tension prestressed tendon in common reinforced concrete pier can increase the recentering capacity of pier and decrease residual displacement of pier under earthquake excitation. And because of the low energy dissipation, it would also increase the displacement demand. The major factors that affect the seismic behavior of integral cast in situ prestressed bridge pier include tension degree and location and reinforcement ratio of prestressed tendon.

In this paper, we study the hysteretic behavior and seismic characteristics of prestressed concrete bridge piers with fiber model.

## 2. Fiber Model Analysis Method

**Figure 1:**
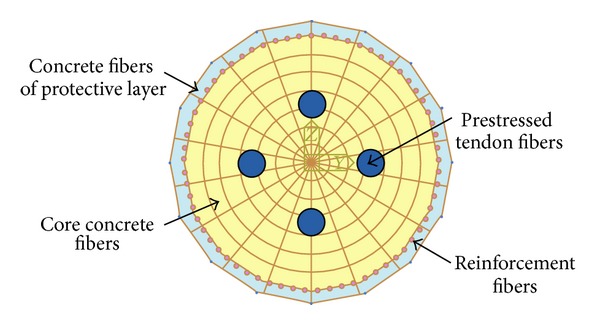
Schematic diagram of fiber section.

The elastic plastic fiber beam elements were adopted to simulate prestressed concrete bridge piers. Elastic plastic fiber beam elements were divided into many sections along the axial, and the central cross section represented the characteristics of each segment, and the cross section was divided into many fibers; the different fibers of the same section had different material properties [2, 9, 12, 13]. As shown in Figure 1, the cross section was divided into enough tiny fiber units, including concrete fiber and reinforced fiber. Prestressed tendons were applied alone without divided fiber units.

*n*_*c*_ is the number of concrete fibers and *n*_*s*_ is the number of reinforced fibers. Related parameters of section fiber table are shown in Table 1.

Based on the assumption of the plane section, the section's resistance at *t* moment is [4, 14]

(1) N=∑ic=1nc{(Et)ic[εt+(ϕx)tyic−(ϕy)txic]Aic}   +∑is=1ns{(Et)is[εs+(ϕx)tyis−(ϕy)txis]Ais} +∑ip=1np{(Et)ip[εt+(ϕx)tyip−(ϕy)txip]Aip},Mx=∑ic=1nc{(Et)ic[εt+(ϕx)tyic−(ϕy)txic]Aicyic} +∑is=1ns{(Et)is[εs+(ϕx)tyis−(ϕy)txis]Aisyis} +∑ip=1np{(Et)ip[εt+(ϕx)tyip−(ϕy)txip]Aipyip},My=∑ic=1nc{(Et)ic[εt+(ϕx)tyic−(ϕy)txic]Aic(−xic)} +∑is=1ns{(Et)is[εs+(ϕx)tyis−(ϕy)txis]Ais(−xis)} +∑ip=1np{(Et)ip[εt+(ϕx)tyip−(ϕy)txip]Aip(−xip)}.

*ε* is axial deformation, *ϕ* is sectional curvature, *N* is axial forces, *M*_*x*_ is moment around the *x*-axis, and *M*_*y*_ is moment around the *y*-axis. Then, the section stiffness is

(2) $$\begin{array}{c} K=\left[\begin{array}{ccc} \overset{n}{\underset{i=1}{\sum }}{\left(E_{t}\right)}_{i}A_{i} & \overset{n}{\underset{i=1}{\sum }}{\left(E_{t}\right)}_{i}A_{i}y_{i} & -\overset{n}{\underset{i=1}{\sum }}{\left(E_{t}\right)}_{i}A_{i}x_{i}\\ \overset{n}{\underset{i=1}{\sum }}{\left(E_{t}\right)}_{i}A_{i}y_{i} & \overset{n}{\underset{i=1}{\sum }}{\left(E_{t}\right)}_{i}A_{i}y_{i}^{2} & -\overset{n}{\underset{i=1}{\sum }}{\left(E_{t}\right)}_{i}A_{i}x_{i}y_{i}\\ -\overset{n}{\underset{i=1}{\sum }}{\left(E_{t}\right)}_{i}A_{i}x_{i} & -\overset{n}{\underset{i=1}{\sum }}{\left(E_{t}\right)}_{i}A_{i}x_{i}y_{i} & -\overset{n}{\underset{i=1}{\sum }}{\left(E_{t}\right)}_{i}A_{i}x_{i}^{2} \end{array}\right]. \end{array}$$

In this formula, *K* is the section's stiffness and *n* = *n*_*c*_ + *n*_*s*_ + *n*_*p*_ is the number of fibers and prestressed tendons.

## 3. The Analysis of PRC Pier's Function under Seismic Performance

**Figure 2:**
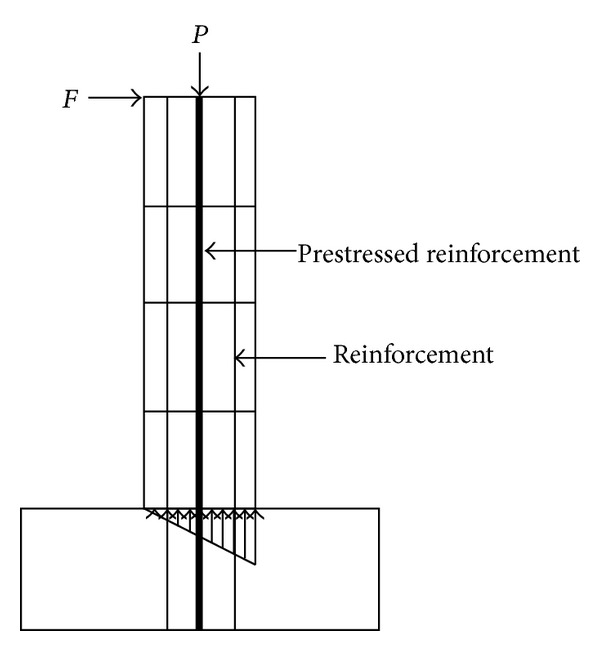
PRC pier.

**Figure 3:**
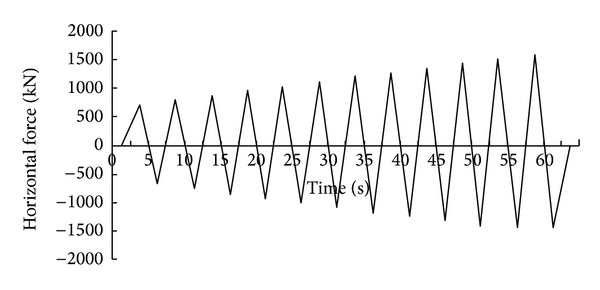
Low-period cyclic loading.

A PRC pier (Figure 2) was 14 m high and the cross-circular section diameter was 2 m. In the horizontal direction, a low cycle loading was applied at the top of pier; the loading method is shown in Figure 3. The peak figure was defined by trial calculation and ensured that the bottom section of the pier had come into the plastic state.

Yield state means that the longitudinal reinforcements at outermost layer of tensile region have been yielded. The ultimate state is that the longitudinal bars have achieved ultimate tensile strain. The software of IDARC was employed in the study.

### 3.1. The Effect of Prestressed Tendon Ratio

Four finite element models were established, which were the same except the number of prestressed tendon. The configuration of prestressed tendon was 5Φ^*s*^15.2, 7Φ^*s*^15.2, 9Φ^*s*^15.2, and 12Φ^*s*^15.2. Accordingly, the ratio of prestressed tendon *ρ*_*p*_ was 0.09%, 0.12%, 0.16%, and 0.21%.

**Figure 4:**
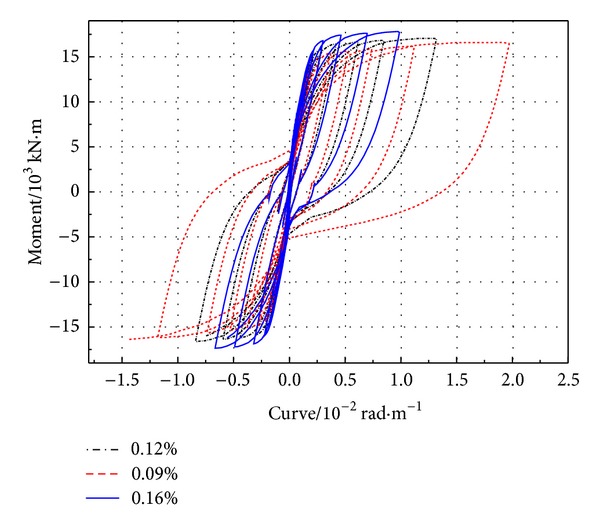
*M* − *φ* curve of bottom section of PRC pier with different prestressed tendon ratios.

**Figure 5:**
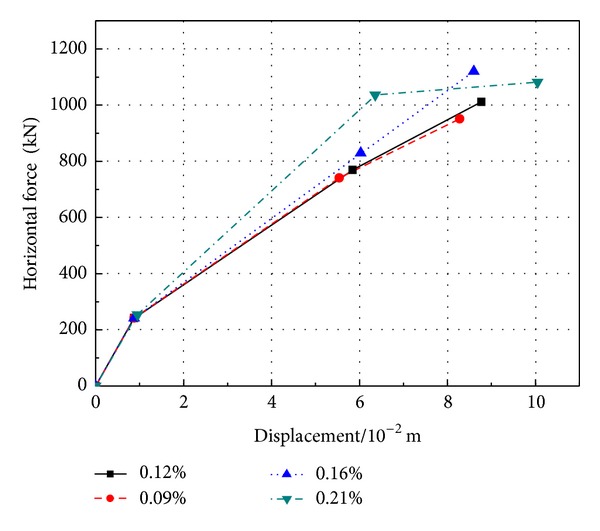
Key points on the skeleton curve of bottom section of PRC pier with different prestressed tendon ratios.

The hysteretic curve (Figure 4) of *M* − *φ* is gotten from the section of plastic hinge area at the bottom of pier. The greater prestressed tendon ratio can lead to more obvious pier's hysteresis loop “pinching effect,” smaller hysteresis loop area, and lower energy dissipation capacity. From Figure 5 and Table 2, it can be obtained that the yield strength improves in some degree that causes the cracking moment and cracking displacement increasing. Generally speaking, PRC pier's ultimate bending moment and ultimate displacement will improve when the ratio of reinforcement increases.

### 3.2. The Influence of the Ratio of Longitudinal Reinforcement

Five finite element models were established; they are all the same except the ratio of longitudinal reinforcement; the configuration of longitudinal reinforcement was 55Φ28, 60Φ28, 70Φ28, 75Φ28, and 80Φ28; the ration of longitudinal reinforcement *ρ*_*l*_ was 1.08%, 1.18%, 1.37%, 1.47%, and 1.57%.

**Figure 6:**
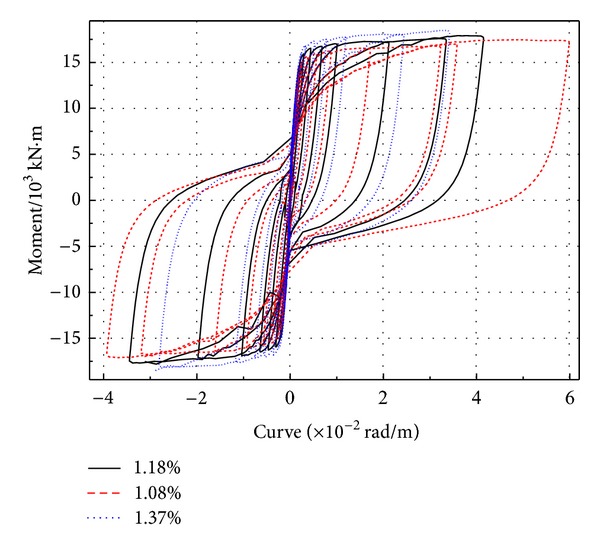
*M* − *φ* curve of bottom section of PRC pier with different longitudinal reinforcement ratios.

**Figure 7:**
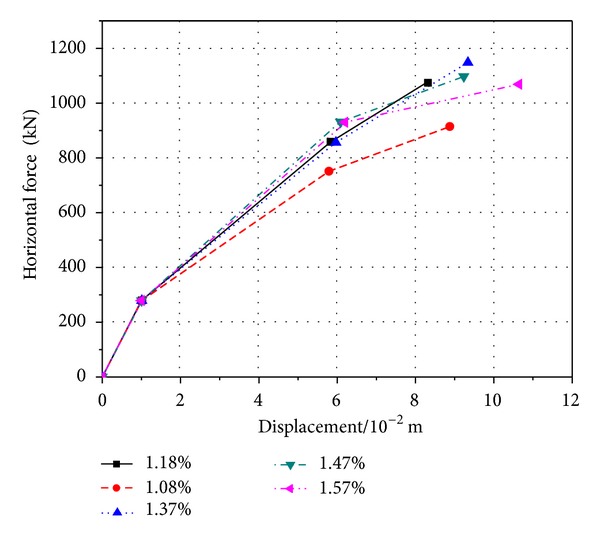
Key points on the skeleton curve of bottom section of PRC pier with different longitudinal reinforcement ratios.

The hysteretic curve (Figure 6) of *M* − *φ* is gotten from the section of plastic hinge area at the bottom of pier. The greater longitudinal reinforcement ratio can make the pier's hysteresis loop “pinching effect” more obvious but smaller. On the other side, the energy dissipation capacity, ductility, and shear capacity will be lower. From Figure 7 and Table 3, it can be obtained that when *ρ*_*l*_ is 1.57%, the yield moment increases to 16.3%, and the ultimate moment increases to 21.6%, compared with *ρ*_*l*_ which is 1.08%. So, the PRC piers' yield stiffness will increase when the ratio of ration of longitudinal bar improves.

### 3.3. The Effect of Stirrup Ratio

We established five finite element models, which are all the same except the ratio of stirrup. The configuration was Φ20@25 cm, Φ20@20 cm, Φ20@15 cm, Φ20@12 cm and Φ20@10 cm. The reciprocal ratio of stirrup *ρ*_*s*_ was 0.08%, 0.10%, 0.13%, 0.17%, and 0.2%.

**Figure 8:**
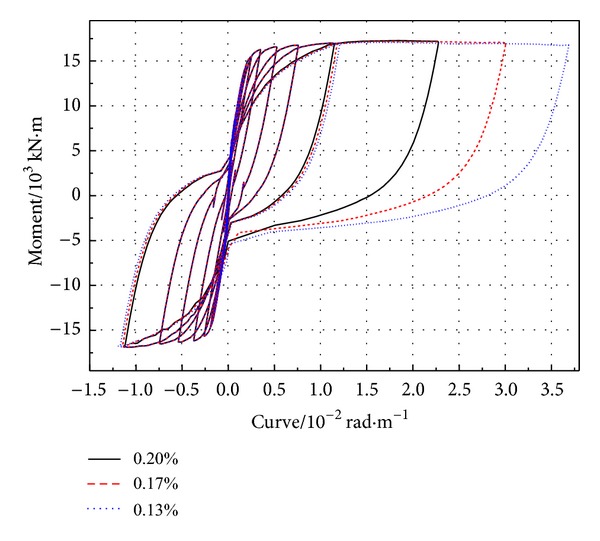
*M* − *φ* curve of bottom section of PRC pier with different stirrups ratios.

**Figure 9:**
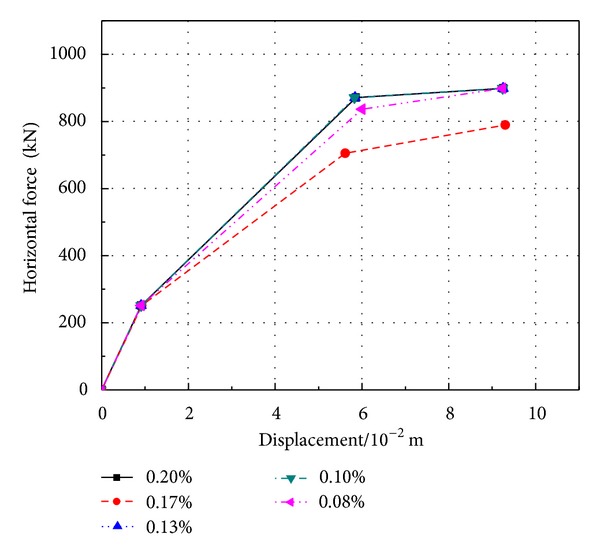
Key points on the skeleton curve of the bottom section of PRC pier with different longitudinal reinforcement ratios.

The hysteretic curve (Figure 8) of *M* − *φ* is obtained from the section of plastic hinge area at the bottom of pier. The greater stirrup reinforcement ratio is, the greater hysteresis loop area is, the better the ductility of bridge piers is, and the stronger the shear capacity is. Because of the increase of the stirrup, the restraint of core concretes may be strengthened and the concrete intensity also is correspondingly enhanced. Furthermore, the nonlinear distortion and the remaining distortion may be reduced. From Figure 9 and Table 4, it can be obtained that when the ratio of stirrup increases, the yield moment and the ultimate moment decrease, and so does the yield displacement.

## 4. Conclusion 

In this paper, the fiber model was adopted to analyze the PRC pier. We studied the hysteretic properties, seismic characteristics, and the related parameters of PRC pier with a low cycle horizontal load applied at the top of pier. The conclusions are drawn as follows.

1. The greater prestressed tendon ratio can lead to more obvious pier's hysteresis loop “pinching effect,” smaller residual displacement, and lower energy dissipation capacity lower. The yield stiffness and ultimate moment increase when the ratio improves in some degree.
2. The greater the longitudinal reinforcement is, the more obvious the pier's hysteresis loop “pinching effect” is and the smaller residual displacement is. In addition, the energy dissipation capacity will be lower and the ductility of bridge piers will be worse.
3. The greater the stirrup reinforcement ratio is, the greater the hysteresis loop area is, the stronger the shear capacity is, and the better the ductility of bridge piers are.

## Figures and Tables

**Table 1:** Characteristics of section fiber.

Type	Number	Coordinate	Area	Tangent modulus
Concrete	*i*_*c*_	(*x*_*i*_*c*__, *y*_*i*_*c*__)	*A*_*i*_*c*__	(*E*_*t*_)_*i*_*c*__
Reinforcement	*i*_*s*_	(*x*_*i*_*s*__, *y*_*i*_*s*__)	*A*_*i*_*s*__	(*E*_*t*_)_*i*_*s*__
Prestressed tendon	*i*_*p*_	(*x*_*i*_*p*__, *y*_*i*_*p*__)	*A*_*i*_*p*__	(*E*_*t*_)_*i*_*p*__

**Table 2:** Calculation results of PRC bridge piers with different prestressed tendon ratios.

*ρ*_*p*_	Yield moment *M*_1_ (kN·m)	Ultimate moment *M*_2_ (kN·m)	*M*_2_/*M*_1_
0.09%	−1.18*E* + 04	−1.47*E* + 04	1.25
0.12%	−1.27*E* + 04	−1.57*E* + 04	1.24
0.16%	−1.35*E* + 04	−1.62*E* + 04	1.20
0.21%	1.47*E* + 04	−1.84*E* + 04	1.25

**Table 3:** Calculation results of PRC bridge piers with different longitudinal reinforcement ratios.

*ρ*_*l*_	Yield moment *M*_1_ (kN·m)	Ultimate moment *M*_2_ (kN·m)	*M*_2_/*M*_1_
1.08%	1.23*E* + 04	−1.53*E* + 04	1.24
1.18%	1.27*E* + 04	−1.53*E* + 04	1.20
1.37%	1.32*E* + 04	1.65*E* + 04	1.25
1.47%	1.38*E* + 04	−1.73*E* + 04	1.26
1.57%	1.43*E* + 04	1.86*E* + 04	1.30

**Table 4:** Calculation results of PRC bridge piers with different longitudinal stirrups ratios.

*ρ*_*s*_	Yield moment *M*_1_ (kN·m)	Ultimate moment *M*_2_ (kN·m)	*M*_2_/*M*_1_
0.08%	1.30*E* + 04	−1.62*E* + 04	1.24
0.10%	1.27*E* + 04	−1.62*E* + 04	1.27
0.13%	1.27*E* + 04	−1.62*E* + 04	1.27
0.17%	1.10*E* + 04	1.36*E* + 04	1.24
0.20%	1.27*E* + 04	−1.61*E* + 04	1.27
